# Chronic cigarette smoke exposure and pneumococcal infection induce oropharyngeal microbiota dysbiosis and contribute to long-lasting lung damage in mice

**DOI:** 10.1099/mgen.0.000485

**Published:** 2020-12-09

**Authors:** Markus Hilty, Tsering M. Wüthrich, Aurélie Godel, Roberto Adelfio, Susanne Aebi, Sabrina S. Burgener, Brunhilde Illgen-Wilcke, Charaf Benarafa

**Affiliations:** ^1^​ Institute for Infectious Diseases, University of Bern, Bern, Switzerland; ^2^​ Institute of Virology and Immunology, 3147 Mittelhäusern, Switzerland; ^3^​ Graduate School for Cellular and Biomedical Sciences, University of Bern, Freiestrasse 1, 3012 Bern, Switzerland; ^4^​ Department of Infectious Diseases and Pathobiology (DIP), Vetsuisse Faculty, University of Bern, 3012 Bern, Switzerland; ^5^​ Microbios, 4153 Reinach, Switzerland

**Keywords:** COPD, emphysema, microbiota, pneumococcus, smoking

## Abstract

Environmental factors, such as cigarette smoking or lung infections, may influence chronic obstructive pulmonary disease (COPD) progression by modifying the respiratory tract microbiome. However, whether the disease itself induces or maintains dysbiosis remains undefined. In this longitudinal study, we investigated the oropharyngeal microbiota composition and disease progression of mice (in cages of 5–10 mice per cage) before, during and up to 3 months after chronic cigarette smoke exposure or exposure to room air for 6 months. Cigarette smoke exposure induced pulmonary emphysema measurable at the end of exposure for 6 months, as well as 3 months following smoke exposure cessation. Using both classical culture methods and 16S rRNA sequencing, we observed that cigarette smoke exposure altered the relative composition of the oropharyngeal microbiota and reduced its diversity (*P* <0.001). More than 60 taxa were substantially reduced after 6 months of smoke exposure (*P* <0.001) However, oropharyngeal microbiota disordering was reversed 3 months after smoke exposure cessation and no significant difference was observed compared to age-matched control mice. The effects of lung infection with *Streptococcus pneumoniae* on established smoke-induced emphysema and on the oropharyngeal microbiota were also evaluated. Inoculation with *S. pneumoniae* induced lung damage and altered the microbiota composition for a longer time compared to control groups infected but not previously exposed to smoke (*P*=0.01). Our data demonstrate effects of cigarette smoke and pneumococcus infection leading to altered microbiota and emphysema development. The reversal of the disordering of the microbiota composition, but not lung damage, following smoke exposure cessation and after clearance of infection suggest that changes in lung structure are not sufficient to sustain a disordered microbiota in mice. Whether changes in the airway microbiota contribute to inducing emphysema requires further investigation.

## Data Summary

The sequence reads from this work have been submitted to the European Nucleotide Archive under accession number PRJEB26965 (https://www.ebi.ac.uk/ena/browser/view/PRJEB26965).

**Impact Statement**

Previous studies have suggested that a disordered lung microbiome is associated with chronic obstructive pulmonary disease (COPD) progression. Whether the disease itself or environmental causative factors, such as cigarette smoke or lung infection, are linked to a disordered microbiome is not known. Therefore, experimental studies with a longitudinal design should be performed. Here, we performed multiple sampling of the oropharyngeal microbiota in mice at 3 month intervals before, during and after chronic exposure (6 months) to cigarette smoke, a model known to lead to emphysema in mice. Since bacterial infections are the most frequent cause for exacerbations in COPD, we additionally evaluated the impact of *Streptococcus pneumoniae* infection on the microbiota composition and disease progression in mice with established smoke-induced emphysema. Overall, we found that: (i) chronic cigarette smoke exposure causes emphysema and alters the composition of the oropharyngeal microbiota, as determined by 16S rRNA sequencing and by bacterial culture; (ii) lung infection with *S. pneumoniae* disrupts lung structure in air- and smoke-exposed mice, and induces a prolonged disordering of the microbiota in the oropharynx in mice previously exposed to cigarette smoke compared to controls; (iii) the alterations in microbiota composition induced by cigarette smoke and by pneumococcal infection are reversed after smoke cessation and after resolution of pneumonia, respectively; (iv) an established emphysema after smoking cessation is not sufficient to maintain a disordered oropharyngeal microbiota.

## Introduction

With one billion active smokers worldwide, cigarette smoking remains a leading risk of mortality and disease burden for cardiovascular disease, lung cancer and chronic obstructive pulmonary disease (COPD) [1]. The vast majority of COPD patients are specifically current cigarette smokers or former smokers [2, 3]. Genetic factors contribute to increasing the risk of developing COPD, as shown in association studies based on human genome sequencing and polymorphism analysis [4–8]. Indeed, the absolute risk of COPD is higher among genetically susceptible smokers [4]. Experimental mouse models have also emphasized the importance of genetic background in the extent of destruction of the lung parenchyma induced by cigarette smoke exposure [9, 10]. Yet, neither smoking status nor genetic profiling alone nor combined can accurately predict whether an individual will develop COPD, suggesting additional contributing factors.

Recent studies point to the local microbiome in the airways and the lungs as potentially associated with disease progression and severity in lung diseases such as pulmonary fibrosis, cystic fibrosis, asthma and COPD [11–14]. As for the latter, a number of studies additionally confirmed significant host–microbiome interactions in COPD revealing associations between the microbial composition, airway inflammation and exacerbations [15, 16]. Cigarette smoke is thought to influence the microbiome composition through immunomodulation [17–20], oxygen deprivation [21], increased bacterial biofilm formation [22, 23] and other mechanisms [24]. Analyses of bronchoalveolar lavage have shown smoking-induced differences in the mouth and oropharynx, but not in the lower respiratory tract microbiome of healthy human subjects [13, 25]. Longitudinal and functional studies are needed to ascertain relationships between altered microbiome, lung disease, genetics and environmental insults such as smoking [26].

It is known that airway infections are most often associated with disease exacerbations leading to a precipitous drop in lung function. Wang and colleagues identified *Haemophilus* and *Moraxella* as potential indicator genera in stable colonization and exacerbation related episodes, respectively [15]. Shared decreased activity of several innate immune defence mechanisms has been described for smoking individuals and COPD patients (see the paper by Sethi and Murphy [27] for more detailed information). The ‘Vicious Circle Hypothesis’ describes a spiralling process of ‘smokeborne’ airway infections that induce inflammatory reactions and impair innate lung defence mechanisms. This self-fuelling process favours bacterial persistence and proliferation, which increase susceptibility to further smokeborne infection events [27, 28], and can lead to severe and recurrent pneumonia. Recent reports have additionally stressed the importance of localized infection foci within the lung parenchyma that may promote COPD progression [29, 30]. *Streptococcus pneumoniae* is a major causative pathogen of community-acquired pneumonia with 27 % of cases [31]. Smokers are at particularly higher risk of acquiring and developing *S. pneumoniae *-induced lung infection, as well as invasive pneumococcal disease, and they have worse outcome predictions as opposed to non-smoking patients [32]. However, little experimental data is available to link cigarette smoke exposure with the respiratory tract microbiome and how infection may contribute to dysbiosis and progression of emphysema.

To investigate these interactions, we performed a longitudinal study where we characterized changes in the upper airway (oropharyngeal) microbiota over time in a chronic (6 month) cigarette-smoke-induced emphysema model in mice using 16S rRNA sequencing and bacterial cultures. Since bacterial infections are the most frequent cause for exacerbations in COPD, we further assessed the impact of *S. pneumoniae* infection on the microbiota composition in mice with established smoke-induced emphysema. By instillation of different *S. pneumoniae* strains in smoke-exposed and control air-exposed mice, we were able to detect dynamic changes of the oropharyngeal microbiota during the course of a self-resolving multifocal pneumonia. Overall, we determined whether infection altered the course of emphysema and whether emphysema contributed to the maintenance of a disordered microbiota.

## Methods

### Experimental set-up and cigarette smoke exposure

**Fig. 1.**
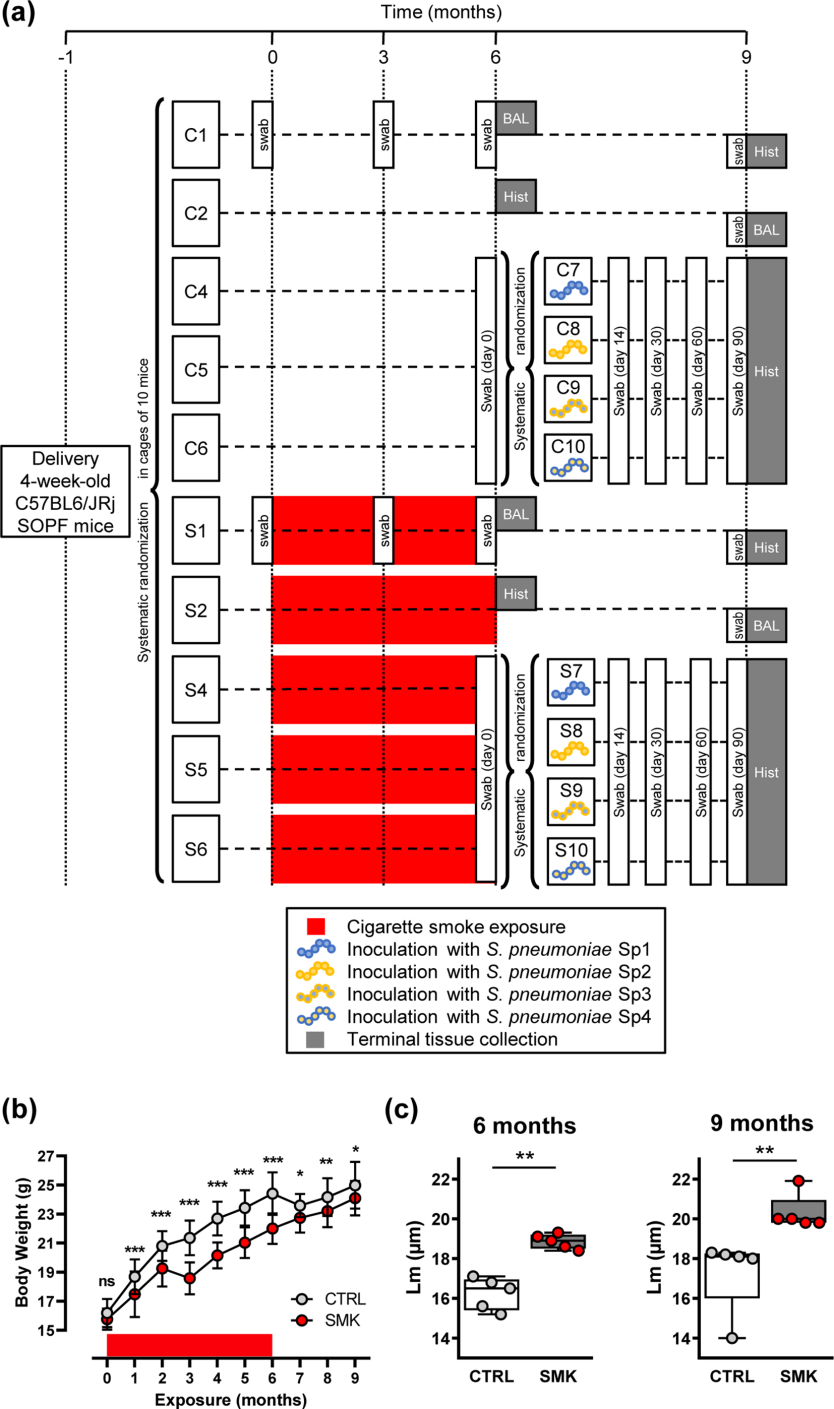
Cigarette smoke induces irreversible emphysema. (a) Experimental scheme showing randomization of purchased female mice into cages of 5–10 mice. After a 4 week acclimation period, the experiment was initiated (time point 0). Groups of mice were exposed to cigarette smoke (cages S1, S2, S4, S5 and S6) or to room air (cages C1, C2, C4, C5, C6) for 6 months. Red boxes represent exposure to cigarette smoke. Note that smoke exposure was stopped at the 6 month time point for all groups. After 6 months of exposure, three sub-groups of control mice (C4, C5 and C6) and smoke-exposed mice (S4, S5 and S6) were further systematically randomized into four groups per exposure regimen and inoculated with 2×10^6^ c.f.u. *S. pneumoniae *. The different strains were Sp1 (106.66 wild-type, serotype 6B), Sp2 (208.41 wild-type, serotype 7F), Sp3 (106.66 capsule mutant cps208.41, serotype 7F) and Sp4 (208.41 capsule mutant cps106.66, serotype 6B). The times of sampling of the microbiota with oropharyngeal swabs are shown. Analysis of lung structure (Hist) or BAL was performed in sub-groups of CTRL and SMK mice that were euthanized at 6 and 9 month time points. (b) Body weight was measured monthly. Data are shown as mean±sd (*n*=50 CTRL; *n*=55 SMK) and were analysed by two-way ANOVA (*** *P*<0.001, ** *P* <0.01, * *P* <0.05, ns (not significant). (c) Structural analysis of the lung parenchyma was performed at the indicated time points. Scatterplots show data for individual mice; median and interquartile range (box) are shown. Data were analysed by Mann–Whitney U test (** *P* <0.01).

Wild-type female C57BL/6J mice were purchased from Janvier Labs at the age of 4 weeks with a certified specific and opportunistic pathogen free (SOPF) status, and were left to acclimatize for 4 weeks. Upon delivery, mice were randomized in groups of 5–10, ear marked and given a unique identification number in a mouse database (TierBase). From the start, groups of mice were randomly assigned to exposure regimen (cigarette smoke or room air) and to the different sample harvest protocol, infection group and timeline. Fig. 1(a) describes treatment and sampling at the cage level for control (cages C1–C10) and smoke-exposed (cages S1–S10) mice. Mice were housed in individually ventilated type II long or type III cages (blue line; Tecniplast), with a 12 h light/dark cycle. Mice were fed *ad libitum* with autoclaved maintenance mouse chow (Kliba) and drank autoclaved acidified tap water. Only female mice were used to avoid potential animal welfare issues arising due to fighting in male mice and inability to isolate aggressive or injured animals in a long-term study.

3R4F reference cigarettes (University of Kentucky, Lexington, KY, USA) were stored at 4 °C and placed at room temperature into a closed container containing a glycerine/water mixture to maintain 60 % relative humidity 2 days before use. Smoke was generated by a microprocessor-controlled Teague TE10z machine (Teague Enterprises) producing a mixture of mainstream (11 %) and side-stream (89 %) cigarette smoke and connected to whole-body exposure chambers to model second-hand exposure to cigarette smoke. Total suspended particulate density was maintained at 100±6 mg m^−3^. On the first week of exposure, mice were gradually adapted by exposure for 30 min on the first day and then for additional 30 min increments every day. Every 90 min exposure was followed by a 60 min break where mice were exposed to room air. From the second week of exposure, mice were exposed for two sessions of 90 min separated by a break of 60 min, 5 days per week for 6 months.

### Experimental set-up for the pneumococcus infection model

Groups of mice exposed to cigarette smoke (cages S4–S6) or air (cages C4–C6) for 6 months were randomly picked in a systematic manner and reassembled into corresponding subgroups (S7–S10 or C7–C10) to be inoculated with one of four different strains of *S. pneumoniae* (Fig. 1a). Sp1 and Sp2 are clinical isolates 106.66 (serotype 6B) and 208.41 (serotype 7F), respectively. Sp3 and Sp4 are capsule switch mutants of these strains 106.66cps208.41 (7F) and 208.41cps106.66 (6B), respectively [33, 34]. For inoculation, mice were anesthetized with ketamine and xylazine (100mg/kg and 10mg/kg, respectively), and intranasally inoculated by pipetting 20 µl onto the nares. Bacterial inoculum was determined as 2×10^6^ c.f.u. per mouse, a dose that we have previously shown to induce a multifocal self-resolving pneumonia and a prolonged colonization of the nasopharynx in C57BL/6 mice exposed to cigarette smoke [35].

### Collection of oropharyngeal swabs

The oropharyngeal microbiota was sampled using ultrafine sterile flock swabs (HydraFlock, 25–3318 h; Puritan). Mice were anesthetized with isoflurane inhalation (4%) and handled in a biosafety cabinet. Swabs were orally inserted and gently rubbed on all sides of the pharynx. Each mouse was sampled twice: the tip of the first swab was cut into a molecular-grade sterile 2 ml tube and stored at −70 °C until DNA extraction was carried out; the tip of the second swab was used to inoculate two blood agar plates and two Columbia plates. To monitor for contamination, multiple control swabs were also processed for DNA extraction and bacterial culture at each throat swab collection.

### Bacterial culture

Duplicate blood and Columbia agar plates were incubated at 37 °C under aerobic or anaerobic conditions using accredited techniques (MicroBioS). Bacterial species were identified based on morphology and the abundance of each bacterial species was determined by colony counting. Control swabs were all negative.

### Lung processing and analysis of lung structure

Stereological analysis of the lungs was performed as we reported previously and according to the American Thoracic Society/European Respiratory Society guidelines [36, 37]. Mice were euthanized with carbon dioxide and lungs were inflated at a constant pressure of 20 cm H_2_O with a buffered fixative solution (1.5 % paraformaldehyde, 1.5 % glutaraldehyde, 0.15 M HEPES, pH 7.35) through a tracheal cannula. The trachea was ligated, and lungs were dissected and stored in fixative for at least 24 h. Total lung volume was determined by the water displacement method. Lungs were embedded in 2.5 % agar and sliced transversally. Systematic, uniform random sampling was used to pick five lung pieces representative of the whole organ and these pieces were embedded on a same plane in paraffin blocks. Lung sections (4 µm) were stained with haematoxylin and eosin. Approximately 50 micrographs at a final magnification of ×200 were captured over the whole surface of the lung sections for each animal using a ColorView IIIu digital camera (Olympus Soft Imaging Solutions) on a Leica DM RB light microscope. Micrographs were equally and systematically placed in a meander using an automated motorized stage. Pictures were quantitatively analysed by using a test system of lines superimposed over the digital images via the STEPanizer [38]. Line counts intersecting septa were used to calculate the mean linear intercept (Lm).

### Sample preparation for 16S rRNA amplicon sequencing

Sample processing, PCR amplification of the 16S rRNA and paired-end 2×250 bp sequencing (Reagent Kit v2) was performed as recently described and outlined in the Supplementary Material [39]. The concentrations of samples were measured using the DNA 7500 kit with an Agilent 2100 Bioanalyzer (Agilent Technologies). Resulting sequencing reads were analysed using the *dada2* package version 1.5.0 and *workflow* in R version 3.1.2. as described elsewhere and also reported in the Supplementary Material [40]. Statistical analysis not related to sequencing data was performed using Prism 8.0 (GraphPad). The test used and the number of animals/replicates are indicated in corresponding figure legends. *P* <0.05 was considered statistically significant.

## Results

### Cigarette smoke causes irreversible emphysema and temporarily impairs body weight gain

Groups of SOPF C57BL/6JRj female mice were purchased at approximately 4 weeks of age and randomized in two groups (in cages of 5–10 mice per cage). After 1 month of acclimatization, mice were exposed to cigarette smoke (SMK, S) or room air (CTRL, C) for 6 months. After 6 months of exposure, subgroups of mice (C4, C5, C6, S4, S5 and S6) were further randomized and inoculated with *S. pneumoniae *, while others were not inoculated (C1, C2, S1 and S2). All subgroups of mice (inoculated or not) were exposed to room air for 3 months to reach the 9 month time point (Fig. 1a). Exposure to cigarette smoke significantly impaired body weight gain, which was partly normalized following cessation of smoke exposure (Fig. 1b). Subsets of mice were euthanized 6 and 9 months after exposure initiation to evaluate pulmonary emphysema by measuring the Lm. In groups of mice not inoculated with *S. pneumoniae *, the Lm was higher in the SMK group compared to the CTRL group after 6 months of exposure, and remained significantly higher in the SMK group compared to the CTRL group until the final end point, 3 months after smoke cessation (Fig. 1c). Thus, our data indicate that chronic smoke exposure (6 months) induces emphysematous changes in the lung structure that are not reversible.

### Cigarette smoke induces oropharyngeal disordering measured by 16S rRNA sequencing

**Fig. 2.**
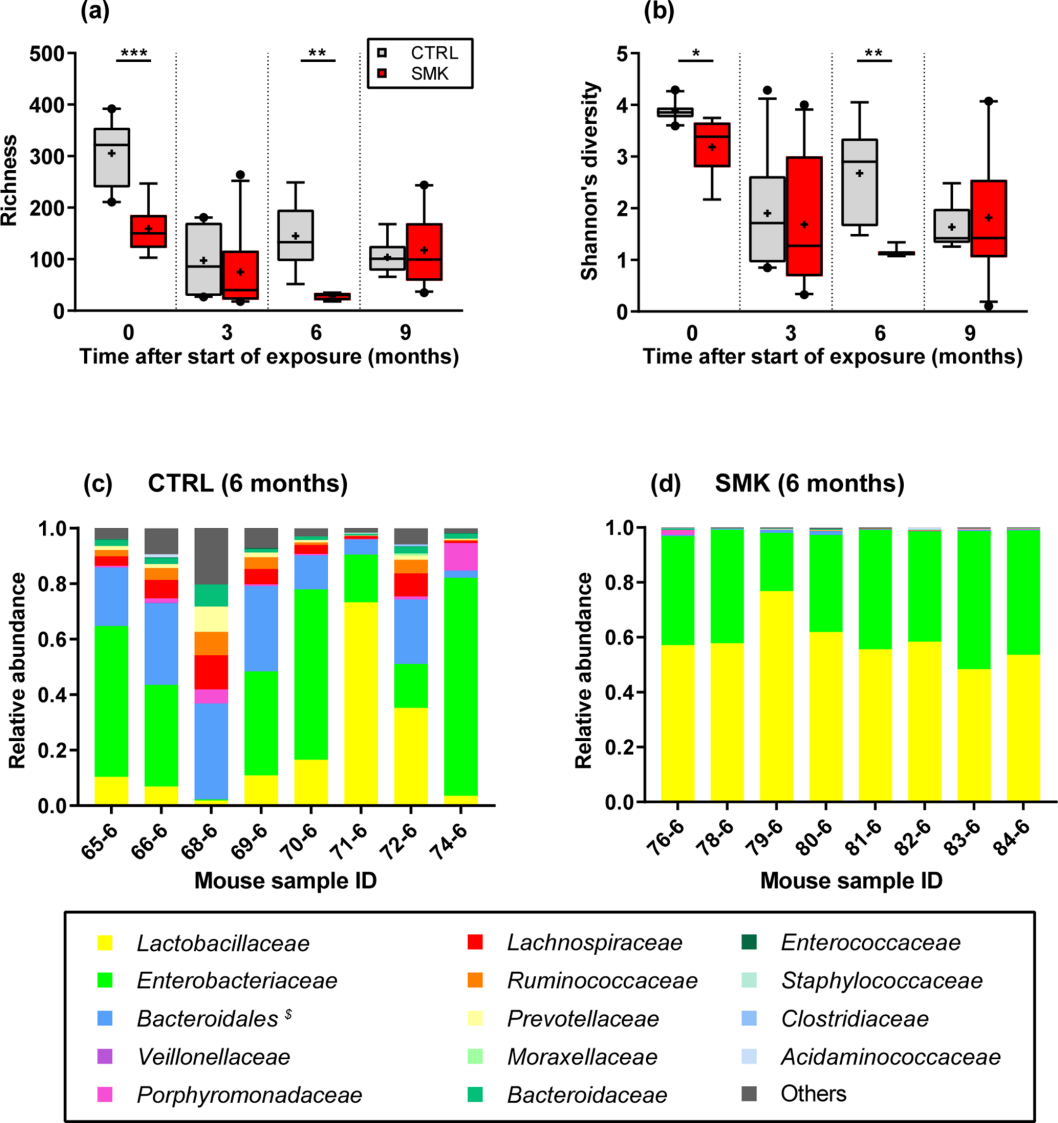
Dynamic changes in oropharyngeal microbiota alpha diversity induced by cigarette smoke. (a) Richness and (b) Shannon’s diversity index are shown for CTRL and SMK mice at the indicated time points (see Fig. 1a). Box-plots indicate median and interquartile range with mean indicated by + and with outliers shown. Data are from 8 to 10 mice per group sampled longitudinally and were analysed by two-way ANOVA (*** *P* <0.001, ** *P* <0.01, * *P* <0.05). (c, d) Bacterial genera identified by 16S rRNA sequencing at the 6 month time point are indicated for individual control (CTRL) (c) and smoke-exposed mice (SMK) (d). *^$^ Bacteroidales* includes all families of the order except *Bacteroidaceae *, which are shown separately.

For the subgroups of non-inoculated mice, the oropharynx was sampled before smoke exposure and at 3 month intervals up to 3 months after the end of exposure (Fig. 1a, animals from cages C1–C2 and S1–S2). Analysing the bacterial density after 16S rRNA amplification revealed no significant differences in smoke-exposed mice as compared to control animals (Fig. S1, available with the online version of this article). We then evaluated the changes in the upper airway microbiota of these mice over time using 16S rRNA sequencing reads. Alpha-diversity analysis showed that smoke exposure for 6 months significantly reduced richness and Shannon’s diversity index, reflecting the number and relative abundance of species identified, respectively (Fig. 2a, b). No significant differences in alpha diversity were visible at the 9 month time point (Fig. 2a, b), indicating that some aspects of disordering can be reversed following cessation of cigarette smoke exposure. Of note, alpha-diversity measurements were significantly different between the groups at the onset (0 month time point). This heterogeneity is likely due to pooling animals from various breeding cages at the vendors’ facility and further randomization upon arrival in our facility. However, using batch correction, we found that very few and rare sequence variants (SVs) (*n*=6) from three families were differentially abundant between the cages at time point zero (*P* value <0.001) (Fig. S2).

At the 3 month time point, we found no statistically relevant differences in alpha diversity between the groups, suggesting that initial differences noted at the basal time point were normalized after further co-housing and that cigarette smoke exposure for 3 months is insufficient to induce measurable changes in alpha diversity. In addition, differential analyses revealed only three SVs having a *P* value <0.001 for the log2 fold changes (see Fig. S3). To further illustrate the observed differences in the alpha-diversity analyses, we plotted the relative abundances of bacterial families at the 6 month time point (Fig. 2c, d). Relative abundances were strikingly altered by cigarette smoke exposure. Most prominently, *Enterobacteriaceae* and *Lactobacillaceae* were increased in the SMK compared to the CTRL group, while there was an almost complete loss of other normally abundant bacterial families (Fig. 2c, d). For the latter, differential analyses revealed 64 SVs being significantly decreased with a *P* value <0.001 for the log2 fold changes between the CTRL and SMK groups (Fig. S4).

**Fig. 3.**
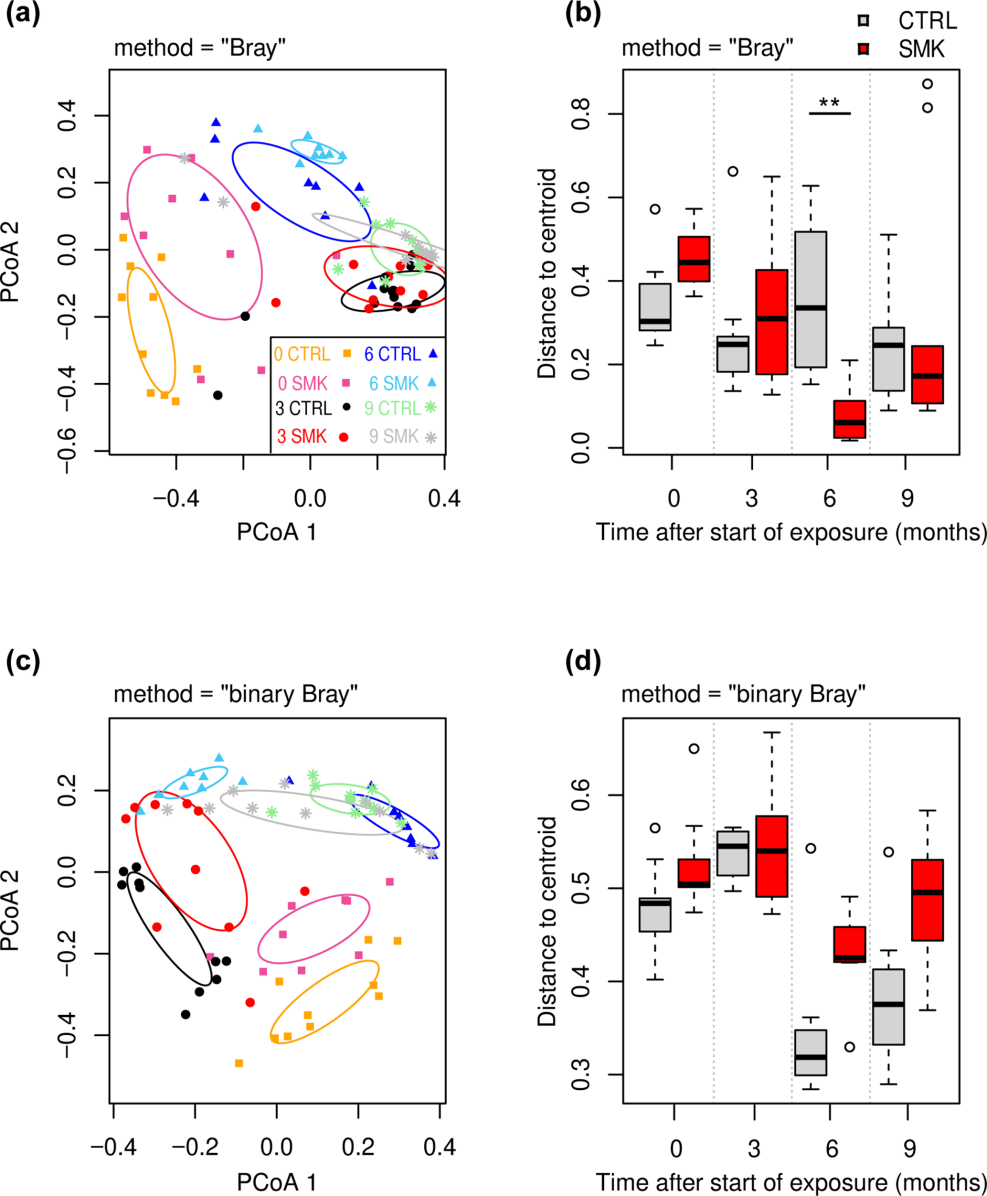
Dynamic changes in oropharyngeal microbiota beta diversity induced by cigarette smoke. (a, b) Weighted Bray–Curtis-based PCoA (a) and corresponding beta-dispersion analysis (b) of the microbial composition at indicated time points for SMK and CTRL groups (Fig. 1a). (c, d) Binary Bray–Curtis-based PCoA (c) and beta-dispersion analysis (d). In PCoA (a, c), symbol shapes indicate time points (TP) (squares, 0; circles, 3; triangles, 6; asterisks, 9 months) with CTRL and SMK group-specific colours described in the key. The 1.0 sd data ellipses are plotted for each group. Excluding the samples for TP=0, PERMANOVA *P* values were *P*=0.004 (time point), *P*=0.2 (smoking status) and *P*=0.5 (smoking status:TP) for data using weighted Bray–Curtis-based PCoA (a). Excluding the samples for TP=0, PERMANOVA *P* values were *P*=0.001 (time point), *P*=0.003 (smoking status) and *P*=0.02 (smoking status:TP) for data using binary Bray–Curtis-based PCoA (a). (b, d) Distances to centroid determined by beta-dispersion analysis are indicated as box-plots with median, interquartile range and outliers shown. Data are from 8 to 10 mice per group sampled longitudinally. The beta-dispersion data were analysed by Mann–Whitney U test (** *P* <0.01).

We further explored the beta-diversity indices of the oropharyngeal microbiota. Principal component analysis (PCoA) based on weighted Ružička distance matrix (DM) (abundance-based) showed that the samples clustered more strongly with the time points rather than exposure (Fig. 3a). A closer observation at each time point reveals that SMK and CTRL groups have minimal overlap with each other at the 0 and 6 month time points (Fig. 3a). Heterogeneity within groups measured by beta-dispersion analysis displayed as distances to centroid revealed a significantly reduced dispersion index after 6 months of smoke exposure (Fig. 3b). Beta-diversity analysis based on binary (presence or absence of SV) DM revealed more profound differences at the 6 month time point between SMK and CTRL groups (Fig. 3c). As in the weighed analysis, binary analysis also revealed a general clustering at each time point independently of smoke exposure, with the notable exception of the SMK 6 month time point, which clustered more closely with SMK 3 month (Fig. 3c). At the 9 month time point, the SMK group samples overlapped in part with the SMK groups at 3 and 6 months and in part with CTRL groups at 6 and 9 months, suggesting gradual reversal of changes induced by cigarette smoke exposure (Fig. 3c). In line, differential analyses of SVs only revealed two SVs being differentially abundant at the 9 month time point (*P* value <0.001) (see Fig. S5). In the binary analysis, distances to the centroids were not statistically different between SMK and CTRL groups (Fig. 3d). Taken together, these findings indicate that chronic exposure to second-hand cigarette smoke in mice alters the airway microbiota as best shown at the 6 month time point using the binary Bray–Curtis PCoA.

### Upper airway bacterial cultures are equally altered by cigarette smoke

**Fig. 4.**
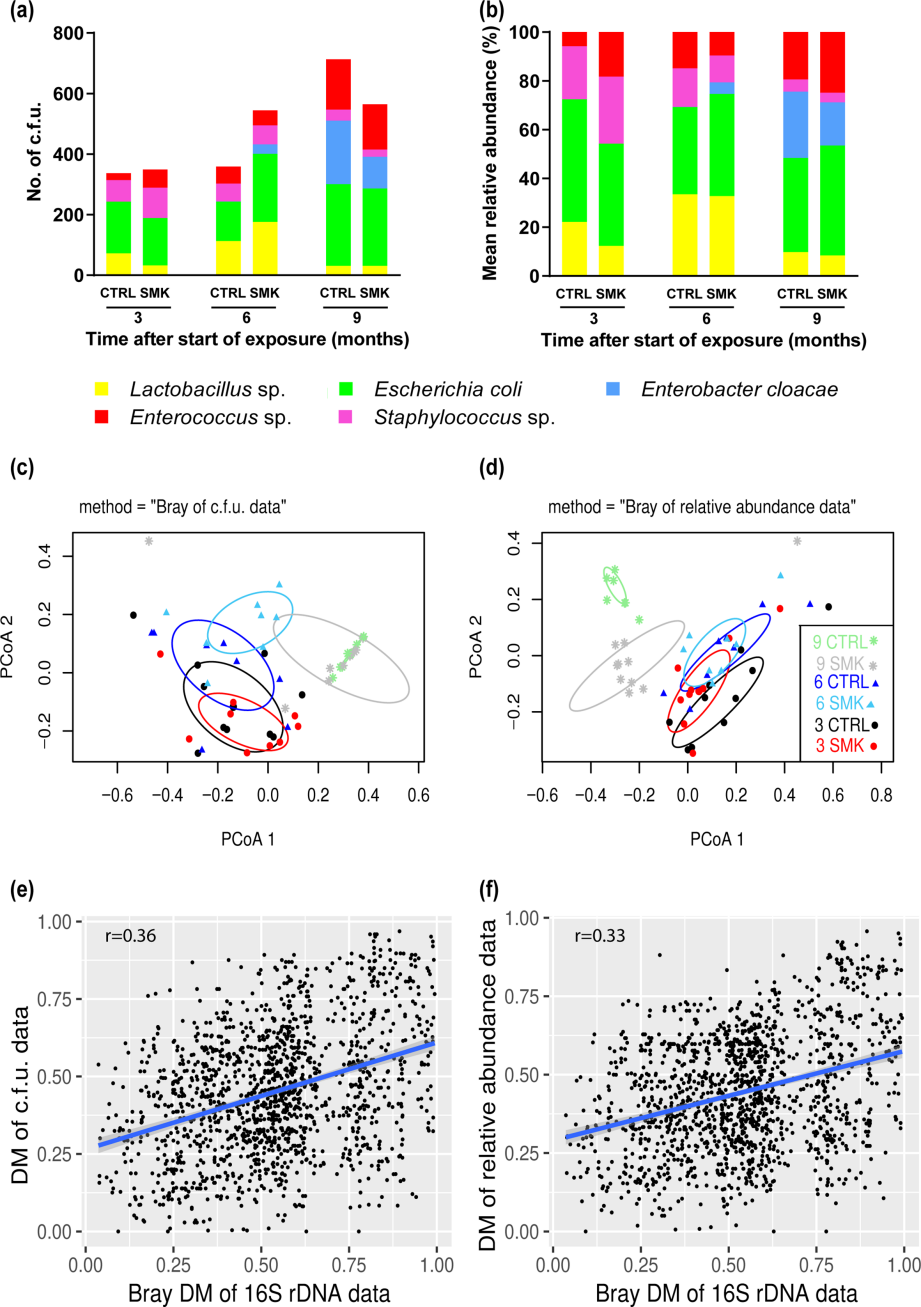
Dynamic changes in oropharyngeal bacterial cultures induced by cigarette smoke. (a, b) Mean absolute numbers of c.f.u. (a) and mean relative abundance (b) of bacterial species cultured from the throat swabs of mice exposed to air (CTRL) or cigarette smoke (SMK) at the indicated time points. (c, d) Bray–Curtis-based PCoA of c.f.u. values (c) and relative abundance (d) of bacterial species are shown for SMK and CTRL mice at the indicated time points. Symbol shapes indicate time points (squares, 0; circles, 3; triangles, 6; asterisks, 9) with CTRL and SMK group-specific colours described in the key. Data are from 8 to 10 mice per group sampled longitudinally. (e, f) Regression analysis between the DMs of the microbiota sequencing data and (e) the bacterial c.f.u. values and (f) the relative abundance of bacterial species.

We also evaluated longitudinal changes in the upper airway microbiota using standard culture techniques in aerobic and anaerobic conditions. The main motivation for the dual analysis was to broadly determine the culturable part of the microbiota, since sequencing techniques are not suitable to do so. The bacterial culture techniques revealed the presence of five major types of bacteria, *Lactobacillus *, *Enterococcus *, *Staphylococcus *, *Enterobacter* and *Escherichia coli *, which were also identified in the sequencing data (Fig. 4a, b). Changes in absolute counts but also in relative abundance of cultured bacteria were observed over time (Fig. 4a, b). Beta-diversity analysis based on weighted DM of absolute counts of bacteria showed effects of smoke exposure after 6 months. In contrast, no differences were detected at the 9 month time point, when SMK mice were not further exposed to cigarette smoke for 3 months (Fig. 4c). When relative abundances rather than absolute counts were used for analysis, differences between exposure groups were noted at 9 months but not at 3 and 6 months after the start of exposure (Fig. 4d). However, beta-diversity analyses generally grouped the samples according to the time points within both analyses (Fig. 4c, d). Finally, weighted DM from the culture and the 16S rRNA sequencing microbiota data were compared and correlation analyses were performed (Fig. 4e, f). Both analyses of cultures using either absolute counts or relative abundance demonstrated significant correlations with the sequencing data (both with *P* <0.001) and *r*=0.36 and *r*=0.33, respectively. Taken together, these findings indicate that the results of sequencing and culture data inform differently on the altered microbiota, yet both approaches also clearly demonstrate similarities and that cigarette smoke exposure significantly modifies the microbiota of the upper airways.

### *S. pneumoniae* infection further increases chronic emphysematous lung injury induced by cigarette smoke

**Fig. 5.**
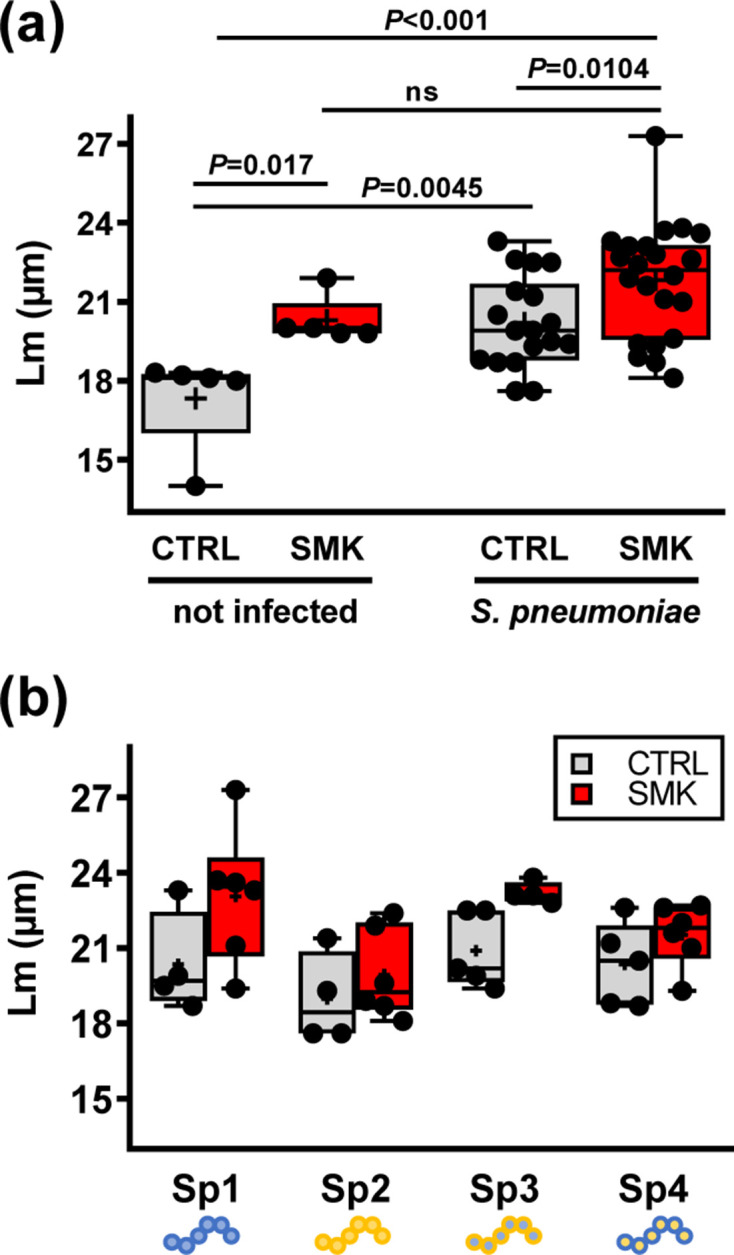
Emphysema in mice exposed to cigarette smoke and pneumococcal infection. Structural analysis of the lung parenchyma was performed at the 9 month time point. (a) Lm of CTRL and SMK groups infected with *S. pneumoniae *; data of the 9 month time point of the first experiment are shown for reference (mock). (b) Lm for individual subgroups infected with strains Sp1 (106.66 wild-type, serotype 6B), Sp2 (208.41 wild-type, serotype 7F), Sp3 (106.66 capsule mutant cps208.41, serotype 7F) and Sp4 (208.41 capsule mutant cps106.66, serotype 6B). Scatterplots show data for individual mice; median, interquartile range (box) and mean (+) are shown. Data were analysed by one-way ANOVA. ns denotes for non-significant

Additional groups of mice that were similarly exposed to cigarette smoke for 6 months were inoculated intranasally with *S. pneumoniae *, a pathogen frequently isolated in patients with progressing COPD (Fig. 1a). No further smoke exposure was applied following infection. Lm measured at 90 days post-infection showed a statistically significant increase in emphysematous changes in *S. pneumoniae *-inoculated SMK mice compared to inoculated CTRL mice (Fig. 5a, *P*=0.01). Importantly, the Lm was increased in infected CTRL mice compared to uninfected CTRL mice at the equivalent 9 month time point (*P*=0.0045), and the Lm was further increased in infected SMK mice compared to uninfected CTRL mice (*P* <0.001) (Fig. 5a). While we did not find a statistically significant difference between the Lm of SMK infected compared to SMK uninfected groups, the overall data suggest that both cigarette smoke exposure and bacterial infection can contribute to disrupt lung structure. Of note, mice were inoculated with either of two different strains 106.66 (Sp1) and 208.41 (Sp2) of *S. pneumoniae* (serotype 6B and 7F, respectively) or with one of the two capsule switch mutants (Sp3, Sp4) [34], where the capsule gene defines the serotype (Fig. 5b). The data suggest strain-specific effects on emphysema development with more noticeable effects for the 106.66 strain (Sp1) and its capsule mutant (Sp3) [33]. However, direct comparisons between strains and with respective mutants were not feasible due to low statistical power.

### Infection-induced changes in upper airway microbiota are sustained by prior cigarette smoke exposure and emphysema

**Fig. 6.**
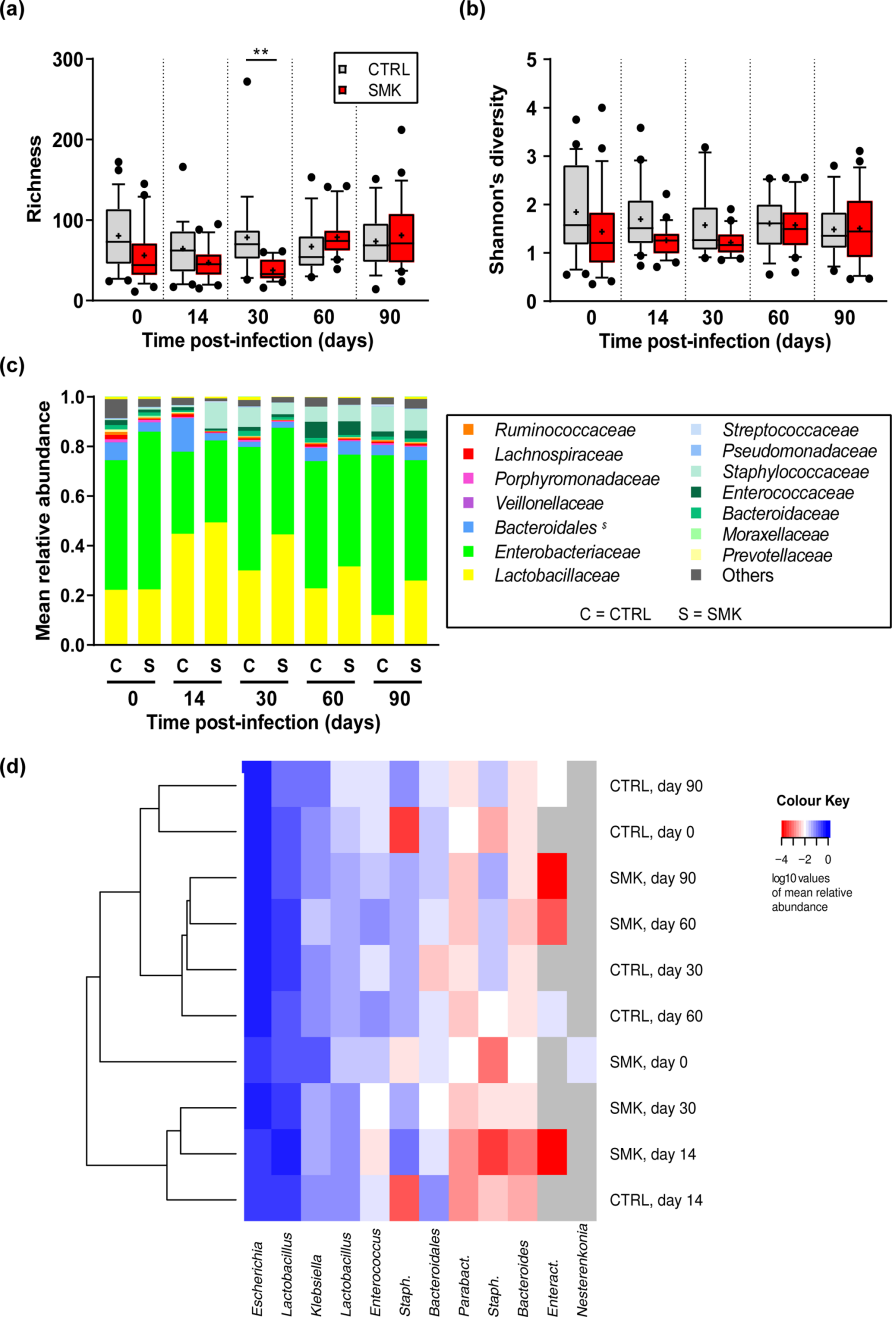
Dynamic changes in oropharyngeal microbiota induced by cigarette smoke and *S. pneumoniae *. (a) Richness, (b) Shannon’s diversity index and (c) mean relative abundance of bacterial genera are shown for CTRL and SMK groups at the indicated time points relative to pneumococcal infection (see the experimental scheme in Fig. 1a). (a, b) Box-plots indicate median and interquartile range with mean indicated as + and with outliers shown. (c) Abundance of bacterial genera identified by 16S rRNA sequencing at each time point show the mean relative abundance for CTRL (C) and SMK (S) at the indicated time points. Data were analysed by one-way ANOVA (** *P* <0.01). (d) Hierarchical clustering analysis of the microbial composition. The mean of the relative abundances of the SVs per sample group has been calculated and the DM created. The dendrogram is based on the DM of all SVs, while the heatmap only visualizes SVs with at least 1 % relative abundance in the respective sample group. All data are from 20 to 24 mice per group sampled longitudinally. SVs are named at the genus level. *Staph*., *Parabact*., and *Enteract* indicate *Staphylococcus *, *Parabacteroides* and *Enteractinococcus *, respectively. *$Bacteroidales* includes all families of the order except *Bacteroidaceae *, which is shown separately.

**Fig. 7.**
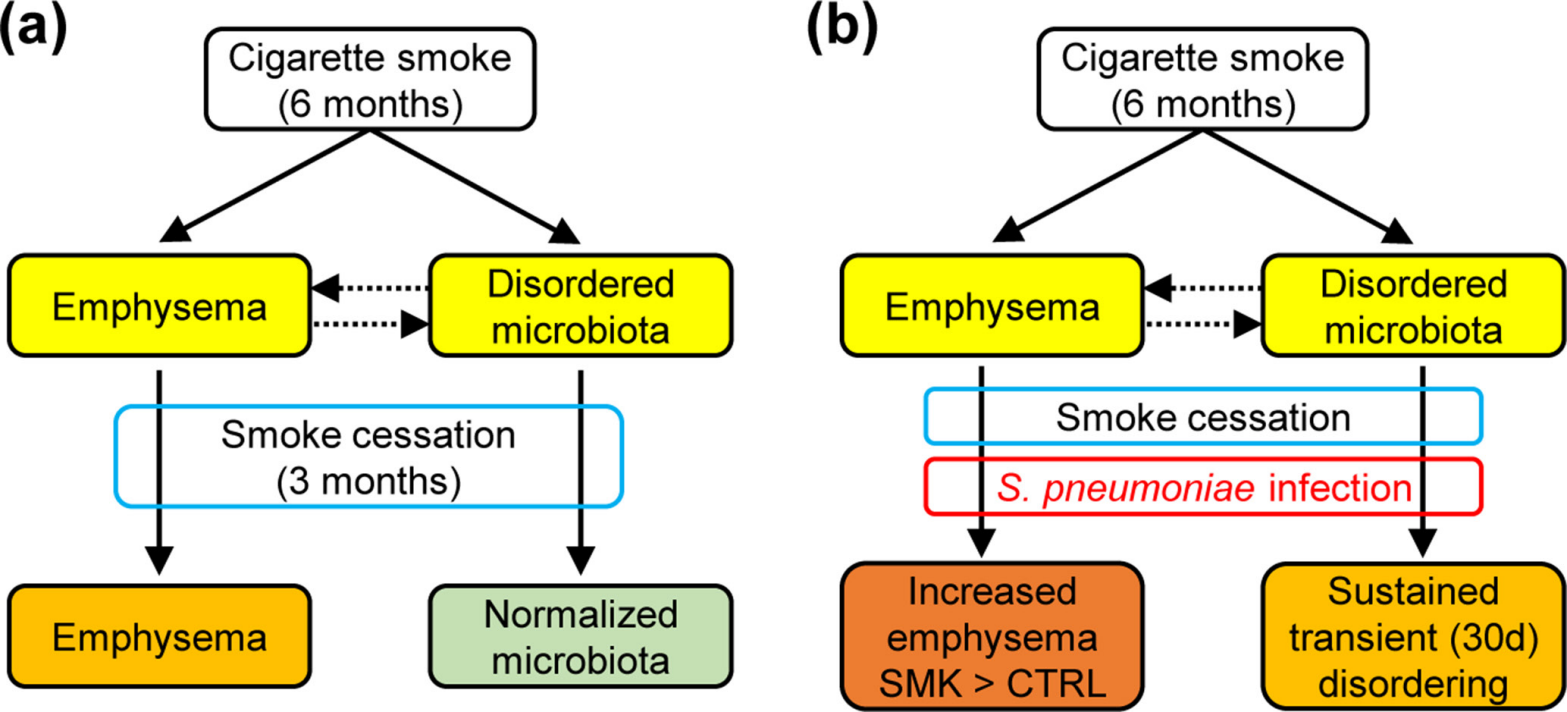
Graphical summary of the combined effects of cigarette smoke and *S. pneumoniae* infection on lung structure and the oropharyngeal microbiota. (a) Chronic cigarette smoke exposure induces irreversible emphysematous changes in lung structure and reversible changes on microbiota composition in the oropharynx. Thus, an established pulmonary emphysema is not sufficient to maintain dysbiosis in the upper respiratory tract. (b) Pneumococcal infection of an established emphysematous lung induces disease exacerbation with long-term consequences on lung structure and a sustained, but reversible, alteration of the oropharyngeal microbiota. Thus, while infection causes structural lung damage, it remains to be established whether a disordered airway microbiota is necessary or sufficient for the development and/or progression of emphysema.

Oropharyngeal swabs were collected after 6 month of exposure and before inoculation (0 days post-infection) and then at multiple time points after inoculation with *S. pneumoniae* (Fig. 1a). The extent of the disordering induced by infection with pneumococcus in SMK and CTRL mice was analysed at the indicated time points post-infection. In both SMK and CTRL mice, infection caused a decrease in diversity at 14 days post-infection (Fig. 6a–c). Importantly, diversity indices were significantly reduced at 30 days post-infection in the SMK group compared to the CTRL group. At 60 and 90 days after infection, richness and Shannon’s diversity index values were increased within each group, indicating that changes due to infection were reversible. Analysis of the relative abundances of bacterial families revealed an increase of *Lactobacillaceae* for both groups at 14 days post-infection and only in the SMK group at 30 days (Fig. 6c). Hierarchical cluster analyses based on the relative abundances of SVs also suggested a distinct clustering for the three sample groups (14 day CTRL, 14 day SMK and 30 day SMK) from the other groups (Fig. 6d). Taken together, our data indicate that chronic cigarette smoke exposure induced both pulmonary emphysema and a disordered microbiota in the upper airways. In addition, smoke exposure cessation lead to a normalization of the microbiota, but defects in lung structure persisted with age even after cessation (Fig. 7a). Thus, an emphysematous lung is not sufficient to maintain a disordered microbiota. Furthermore, our data show that pneumococcal lung infection exacerbated emphysematous changes induced by cigarette smoke, and that smoke exposure and infection synergized to sustain microbiota disordering (Fig. 7b).

## Discussion

Several cross-sectional studies have shown that a disordered lung microbiome is associated with COPD [13, 41]. However, only a few reports included an experimental design that would reveal longitudinal relationships between a changing microbiome and disease phenotypes [15, 42]. We acknowledge that the human respiratory tract microbiome is likely more varied and distinct from the microbial communities of laboratory mice. Yet, mouse models are relevant, controlled tools to investigate temporal changes in the microbiome during disease development. The oropharynx – rather than the lower airways – was preferred here as it allowed multiple, longitudinal sampling of the same mice at multiple time points in the study. Cigarette smoke exposure is also a relevant model inducing chronic lung disease as it is a common causal factor in both humans and mice. Low-biomass microbiome studies specifically require multiple procedural quality controls to rule out contamination [43–45]. In our case, bacterial DNA content of negative control swabs was below the predefined cut-off for sequencing for all specimens. In addition, no colonies were produced from any negative control oropharyngeal swab using standard culture techniques under aerobic and anaerobic conditions. There were also some significant correlations between the different datasets (16S rRNA and culture), further indicating that 16S rRNA sequencing data truly were originating from culturable bacteria and not from reagents used in DNA isolation and library preparation, which can contain contaminating bacterial DNA [44]. The chosen model and methods provide experimental evidence that cigarette smoke exposure and pneumococcal lung infection induce profound but reversible disordering of the oropharyngeal microbiota. To some extent, our conclusions may be extrapolated to better understand human respiratory diseases.

In this study, we first analysed the effects of cigarette smoke exposure on the composition of the oropharyngeal microbiota and on the development of chronic pulmonary disease. The oropharyngeal microbiota composition has been found to correlate better with the lower airway microbiota as compared to other sites such as the nasopharynx [46, 47]. Analysis of the oropharyngeal microbiota using 16S rRNA sequencing revealed major changes induced by cigarette smoke after 6 months of exposure, but not after only 3 months. Compared to the control mice, the abundance of *Lactobacillus* spp*.* was increased and other taxa were significantly decreased in the smoke-exposed mice after 6 months. An earlier study investigated the correlation between smoking and salivary microbe levels in 780 human subjects [48]. Though salivary and oropharyngeal samples are not directly comparable, smoking was strongly associated with higher *Lactobacillus* counts in saliva. Increased proportions of high *Lactobacillus* counts were also reported in the saliva of smokers in a study including an unmedicated adult population of 462 non-smokers and 180 smokers [49]. In addition to increased *Lactobacillus* spp., we also observed a decrease in the values of the alpha diversity after 6 months of cigarette smoke exposure. In a recent study, bronchial wash samples from adults with COPD (*n*=18), smokers with no airway disease (*n*=8) and healthy individuals (*n*=11) were analysed by extended-culture and culture-independent Illumina MiSeq sequencing [50]. As in our study, microbial community composition of patients with COPD was significantly different from that found in smokers and non-smokers, including decreased alpha diversity, suggesting that changes in microbiological status are a component of the pathogenesis.

In our study, we observed that the upper airway bacterial communities of SOPF mice from the same unit of a single provider can be heterogeneous, as illustrated by high alpha diversity even after 1 month of co-housing before the first sampling at day 0. Similarly, no core lung microbiota was found at day 0 in a study comparing lung, tongue and caecal bacteria in 40 healthy, genetically identical, 10-week-old mice, using 16S rRNA quantification and sequencing [44]. Furthermore, increased duration of co-housing then led to a conversion of murine lung bacteria, which we observed also in this study of the oropharynx microbiota with greater similarity between groups at the 9 month time point. Duration of smoke exposure also seems to be critical to observe changes in the microbiota composition. In an experimental mouse model, cigarette smoke exposure for 1 week did not alter the nasal microbiota composition [51]. Similarly, we found that the oropharynx microbiota did not reveal disordered microbial communities after 3 months, but only after 6 months of smoke exposure. These findings indicate that long-term smoke exposure in combination with emphysema development is likely required to induce differences in the microbiota composition. Importantly, we found that cessation of smoke exposure for 3 months led to a reversal of the changes, indicating that normalization of the microbiota may be an additional beneficial effect of smoking cessation. Perhaps as a limitation, we only investigated female mice though the sex is potentially important in lung microbiome research [52].

It is difficult to disentangle the direct effects of long-term smoke exposure on microbiome disordering from those mediated by inflammation. A case for the effects of host response on the microbiome is supported by experimental and epidemiological studies. Mice challenged with lipopolysaccharide (LPS)/elastase intranasally over 4 weeks develop a chronically inflamed and damaged lung, and a decrease in lung microbiota richness and diversity [53]. An increased representation of the genera *Pseudomonas* and *Lactobacillus* and a reduction in *Prevotella* were specifically reported. Although the sampling approach and mouse model in that study differed from ours, both studies showed decreased alpha-diversity values and increased *Lactobacillus* in association with the development of emphysema. Furthermore, smokers without overt lung disease appear to have an unchanged lung microbiota composition as compared to non-smokers [25, 50]. It is tempting to speculate that smoke-induced COPD in humans and emphysema in mice require a prior change in the microbiota, but this remains to be experimentally proven.

Pulmonary infections are important factors in disease exacerbations in advanced COPD. Yet, it is unclear whether such infections contribute further to chronic structural defects and whether they have consequences on the local microbiome. The additive effects of cigarette smoke exposure and of *Streptococcus* infection on emphysema severity, microbiome alteration and colonization were evaluated in mice exposed for 6 months and inoculated with four different pneumococcal strains. We chose serotype 6B and 7F strains, which have been shown to have different pneumococcal capsule thickness due to the difference in the efficiency for the precursor usage for capsule synthesis [34]. We used a relatively high infectious dose that we previously established was non-lethal but yet sufficient to cause pneumonia and a prolonged colonization in mice exposed to cigarette smoke [35]. We found that lung infection worsens the emphysema as measured 90 days after infection and it may also affect lung structure in control mice. In addition, we found that pneumococcal infections induce a more durable dysbiosis in mice previously exposed to smoke. However, these effects appear to be transient and emphysema did not promote a long-term establishment of a modified microbiome. We were not able to conclude on pneumococcal strain-specific differences in part because intra-group variation was higher than anticipated, which reduced the statistical power of the study. However, there was a tendency that the wild-type 106.66 strain of the frequently isolated serotype 6B caused more severe diseases compared to the 208.41 invasive serotype 7F pneumococcal strain. In an earlier study, the ability of identical 6B and 7F wild-type strains and mutants to colonize the nasopharynx of outbred MF1 female mice was investigated [33]. At day 7, a disadvantage of a 7F capsule, but not a 6B capsule, in colonization was apparent. Our data suggest that the capsule type has less importance in enhancing smoke-induced emphysema as the two strains with the 106.66 backbone (Sp1 and Sp3) tended to have higher Lm values regardless of capsule type. Further work is required to investigate these findings. We previously examined the clearance of *S. pneumoniae* and *Pseudomonas aeruginosa* and associated immune responses in mice exposed to cigarette smoke or after smoking cessation [35]. We found that cigarette smoke induced a temporary and reversible increase in the clearance of lung pathogens. This is in line with this study, which suggests that the changes in the microbiota are reversible upon cessation of smoke exposure.

To conclude, we demonstrated a synergistic effect between cigarette smoke exposure, altered microbiota and pneumococcal infection leading to persistent emphysematous changes in the lungs. While smoke-induced and infection-induced microbiota disordering were each reversible upon smoke exposure cessation and infection clearance, there was also an association that the structural changes of the lung were sustained and additively impacted by the two challenges. Whether dysbiosis is necessary and sufficient for emphysema development remains to be proven experimentally and our data suggest that microbiota transfer studies are warranted.

## Supplementary Data

Supplementary material 1
