# Supplementary material for: Chronic cigarette smoke exposure and pneumococcal infection induce oropharyngeal microbiota dysbiosis and contribute to long-lasting lung damage in mice
Source: Microb Genom. 2020 Dec 9;6(12):mgen000485. doi: 10.1099/mgen.0.000485 (PMC8116676; doi:10.1099/mgen.0.000485)

Supplementary material:

Sample preparation for 16S amplicon sequencing. Sample processing was performed as recently described [1]. In brief, the DNA from all samples was extracted using the Qiagen DNA Minikit (Qiagen, Hilden, Germany), following the spin protocol for DNA purification from body fluids. Subsequently, the V4 region of the 16S rRNA gene was amplified using forward (5'-GTGCCAGCMGCCGCGGTAA-3') and reverse (5'-GGACTACHVGGGTWTCTAAT-3') primers [2] and modified with an Illumina adaptor sequence at the 5' end. PCR cycling conditions were 95 °C for 6 minutes and 40 cycles at 95 °C for 30 seconds, at 59 °C for 30 seconds and at 72 °C for 1.5 minutes (with a final elongation step at 72 °C for 5 minutes). PCR products were purified by QIAquick PCR purification kit (Qiagen, Hilden, Germany). The samples were semi-quantified using the DNA 7500 kit with an Agilent 2100 Bioanalyzer (Agilent Technologies, Palo Alto, CA). As suggested previously, samples with less than 1 ng/µl after PCR and purification should be excluded from further analyses [3]. As part of our quality control, four swabs were mock-exposed for several seconds during each sampling procedure and processed together with the samples from this study. All negative control samples were below 1 ng/µl after PCR and purification and, therefore, mock samples were not sent for sequencing. Samples were submitted for indexing and pair-end 2x250 bp sequencing (Reagent Kit v2) on the Illumina MiSeq platform (San Diego, USA).

Inferring sequence variants (SVs) of 16S amplicon sequencing data. Reads were analysed using the *dada2* package version 1.5.0 and *workflow* in R version 3.1.2. as previously described [4] In brief, forward and reverse reads were trimmed at 200 bp and at 150 bp to remove low quality regions, respectively. The amplicon errors were corrected using the *dada2* algorithm with default parameters. SVs shorter than 245 or longer than 257 base pairs where removed as were chimeras. Taxonomy was assigned using the *assignTaxonomy* function [5].

Alpha and beta diversity analyses of SVs (for the non-infection analysis). Alpha diversity was assessed with the functions *estimate* (richness) and *diversity* (Shannon diversity indices [SDIs]). In brief, the richness was calculated using the command *specnumber* in R, and the SDI via the *diversity* function. As for analysing the SVs, the distance matrices (DM; square matrix containing the distances, taken pairwise between two different samples) for Beta diversity analyses were calculated by unweighted Jaccard index (presence-absence based) and weighted Ružička index (abundance-based) of dissimilarity, and the distances were calculated using the *vegdist* function from the vegan package. The multivariate dispersion of each sample group was determined by calculating the average distance (based on Jaccard and Ružicka indices) to the sample type's centroid using the *betadisper* function. Outputs were visualised in principal component analyses (PCoA) (*plot* function) and beta dispersion boxplots (*boxplot* function).

For the purpose of additional differential analysis, amplicon SVs were inferred with dada2 1.16.0 and contaminating sequences across the entire data set were identified with decontam 1.8.0 using comparison to sequence prevalences in a set of negative controls that were collected at the same time

and treated the same way as the samples. Batch effect correction between cages was carried out separately for each timepoint by batch mean centering (BMC) on centered log-ratio transformed (CLR) abundance data. The corrected data sets underwent differential analysis for each time point separately with the R package DESeq 1.28.1. (model: ~ cage + group, test: Wald).

A particular strength from our study is the parallel inclusion of culture data. Species from culture were identified and semi-quantitatively counted. The resulting numbers were transferred to absolute and relative values for comparison to sequencing data. Furthermore, we calculated the DM for absolute (CFUs) and relative values by the weighted Ružička index (abundance-based analyses) using the vegdist function. We then computed the PCoA plots as described for the SVs above. Linear correlation of paired dissimilarity value of weighted (Ružicka) and culture data (absolute or relative values) DM were calculated using *aes* and *geom_smooth* (method = "lm") function of ggplot2 in R.

Alpha and beta diversity analyses of SVs (for the infection analyses). Alpha diversity values of the animals which were inoculated by any of the 4 different pneumococcal strains were pooled. Relative abundances of bacterial families were also pooled and visualised as average relative abundances per time point.

As for Beta diversity analyses average relative abundances of each SV for each of the four groups was first calculated. Subsequently, we received the DM by weighted Ružička index (abundance-based) calculation (vegdist function) and clustered the results to receive a dendrogram (*hclust* function). Results were then visualised by a dendrogram (function as. Dendrogram) and a heat map of the most abundant SVs (log10 values) using the heatmap.2 function and ggplot2 package in R.


1. **Kraemer JG, Ramette A, Aebi S, Oppliger A, Hilty M**. Influence of pig farming on the human's nasal microbiota: The key role of the airborne microbial communities. *Appl Environ Microbiol* 2018.
2. **Caporaso JG, Lauber CL, Walters WA, Berg-Lyons D, Lozupone CA et al.** Global patterns of 16S rRNA diversity at a depth of millions of sequences per sample. *Proc Natl Acad Sci U S A* 2011;108 Suppl 1:4516-4522.
3. **Biesbroek G, Sanders EA, Roeselers G, Wang X, Caspers MP et al.** Deep sequencing analyses of low density microbial communities: working at the boundary of accurate microbiota detection. *PLoS One* 2012;7(3):e32942.
4. **Callahan BJ, McMurdie PJ, Rosen MJ, Han AW, Johnson AJ et al.** DADA2: High-resolution sample inference from Illumina amplicon data. *Nat Methods* 2016;13(7):581-583.
5. **Wang Q, Garrity GM, Tiedje JM, Cole JR**. Naive Bayesian classifier for rapid assignment of rRNA sequences into the new bacterial taxonomy. *Appl Environ Microbiol* 2007;73(16):5261-5267.

**Supplementary figure legends**

**Supplementary Figure 1: Bacterial density in the oropharynx of mice.** PCR concentrations were measured by the Bioanalyzer and are shown for CTRL and SMK exposed mice at indicated time points. Box-plots indicate median and interquartile range with mean indicated as + and outliers are shown. Data are from 8-10 mice per group sampled longitudinally

**Supplementary Figure 2: Differential abundance of SVs in SMK and CTRL groups prior to smoke exposure.** DESeq analysis indicates the fold-change of SVs in bacterial communities of mice prior to the start of smoke or air exposure ($p < 0.001$). Mice were assigned in systematic random manner into cages and acclimatized for 4 weeks prior to the first oropharyngeal swab sampling (time point 0) taken before the initiation of exposure to cigarette smoke or room air. The differential abundances of SVs between SMK and CTRL groups with $p<0.001$ only are presented. The taxa of the SVs are labeled at genus level and the family belongings are indicated in the identical colors as in Figure 2C and 2D. The data shown here includes bacterial families that were regrouped as 'others' and shown in grey in in Figure 2C and 2D.

**Supplementary Figure 3: Differential abundance of SVs in SMK and CTRL groups after three months.** DESeq analysis indicates the fold-change of SVs in bacterial communities of mice after three months of smoke or air exposure ($p < 0.001$). The differential abundances of SVs between SMK and CTRL groups with $p<0.001$ only are presented. The taxa of the SVs are labeled at genus level and the family belongings are indicated in the identical colors as in Figure 2C and 2D. The data shown here includes bacterial families that were regrouped as 'others' and shown in grey in in Figure 2C and 2D.

**Supplementary Figure 4: Differential abundance of SVs in SMK and CTRL groups after six months.** DESeq analysis indicates the fold-change of SVs in bacterial communities of mice after six months of smoke or air exposure ($p < 0.001$). The differential abundances of SVs between SMK and CTRL groups with $p<0.001$ only are presented. The taxa of the SVs are labeled at genus level and the family belongings are indicated in the identical colors as in Figure 2C and 2D. The data shown here includes bacterial families that were regrouped as 'others' and shown in grey in in Figure 2C and 2D.

**Supplementary Figure 5: Differential abundance of SVs in SMK and CTRL groups after nine months.** DESeq analysis indicates the fold-change of SVs in bacterial communities of mice after nine months ($p < 0.001$). One group of mice were smoke exposed for 6 months followed by a period of 3 months of smoke cessation (9 months in total) (SMK) while the control mice were air exposed for the whole time for 9 months (CTRL). The differential abundances of SVs between SMK and CTRL groups with $p<0.001$ only are presented. The taxa of the SVs are labeled at genus level and the family belongings are indicated in the identical colors as in Figure 2C and 2D. The data shown here includes bacterial families that were regrouped as 'others' and shown in grey in in Figure 2C and 2D.

# Supplementary figure 1

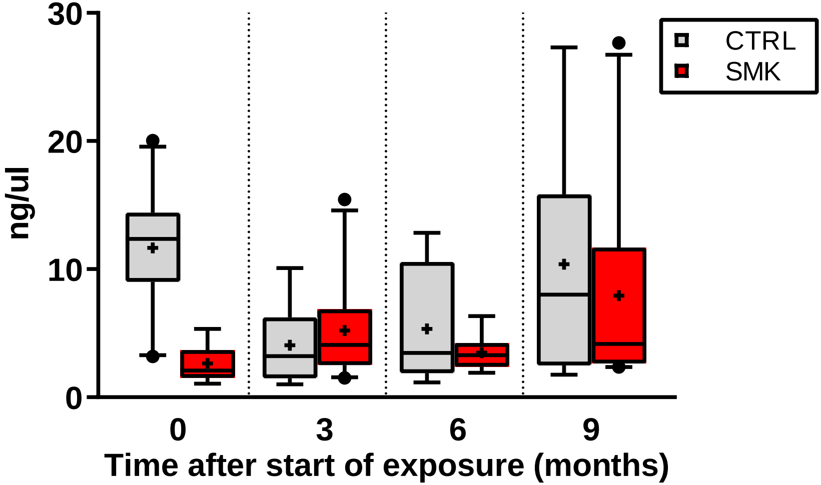

Supplementary figure 2: Differential abundance at time point 0 (adj. p-value = 0.001)

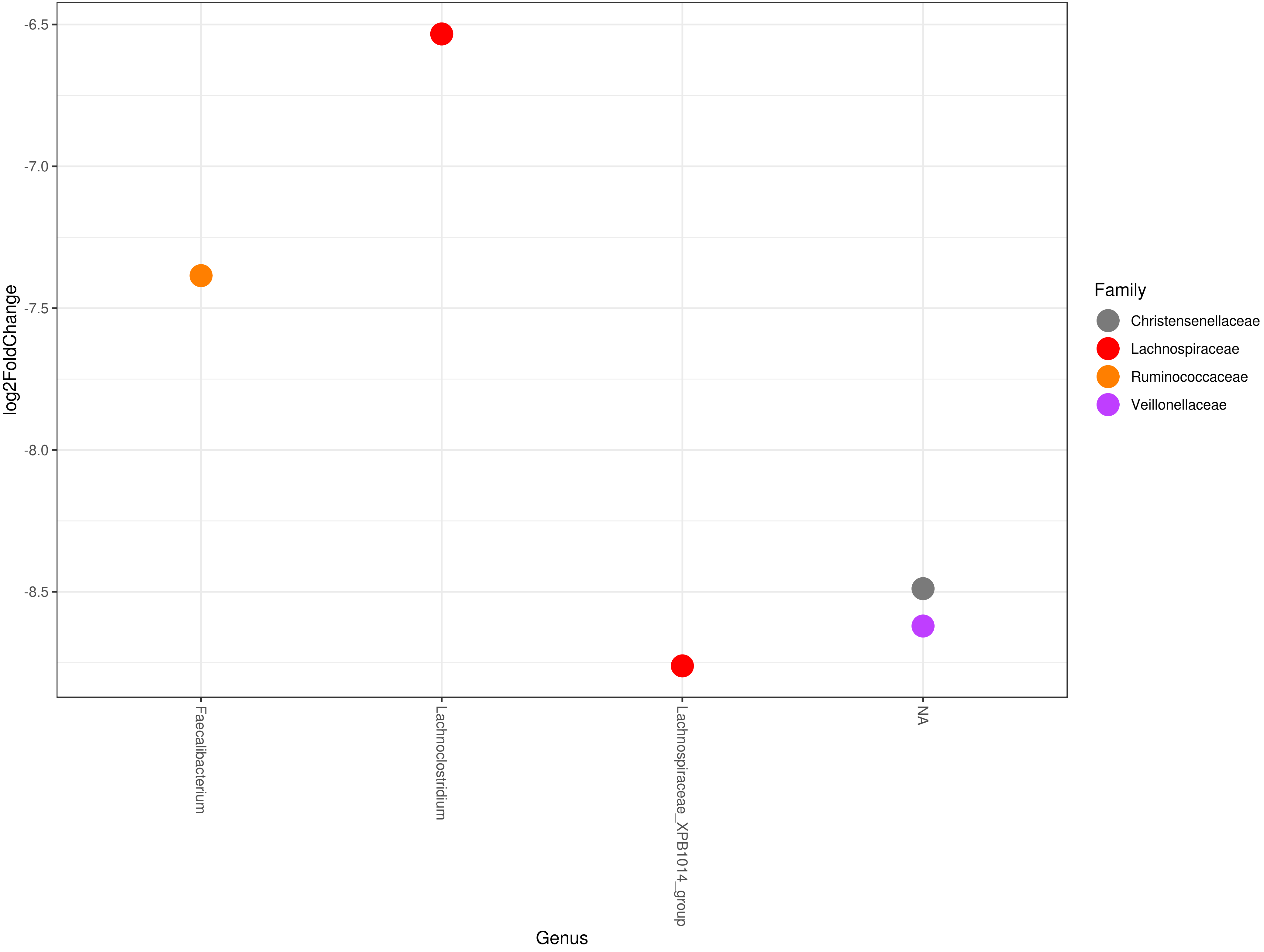

Supplementary figure 3: Differential abundance at time point 3 months (adj. p-value = 0.001)

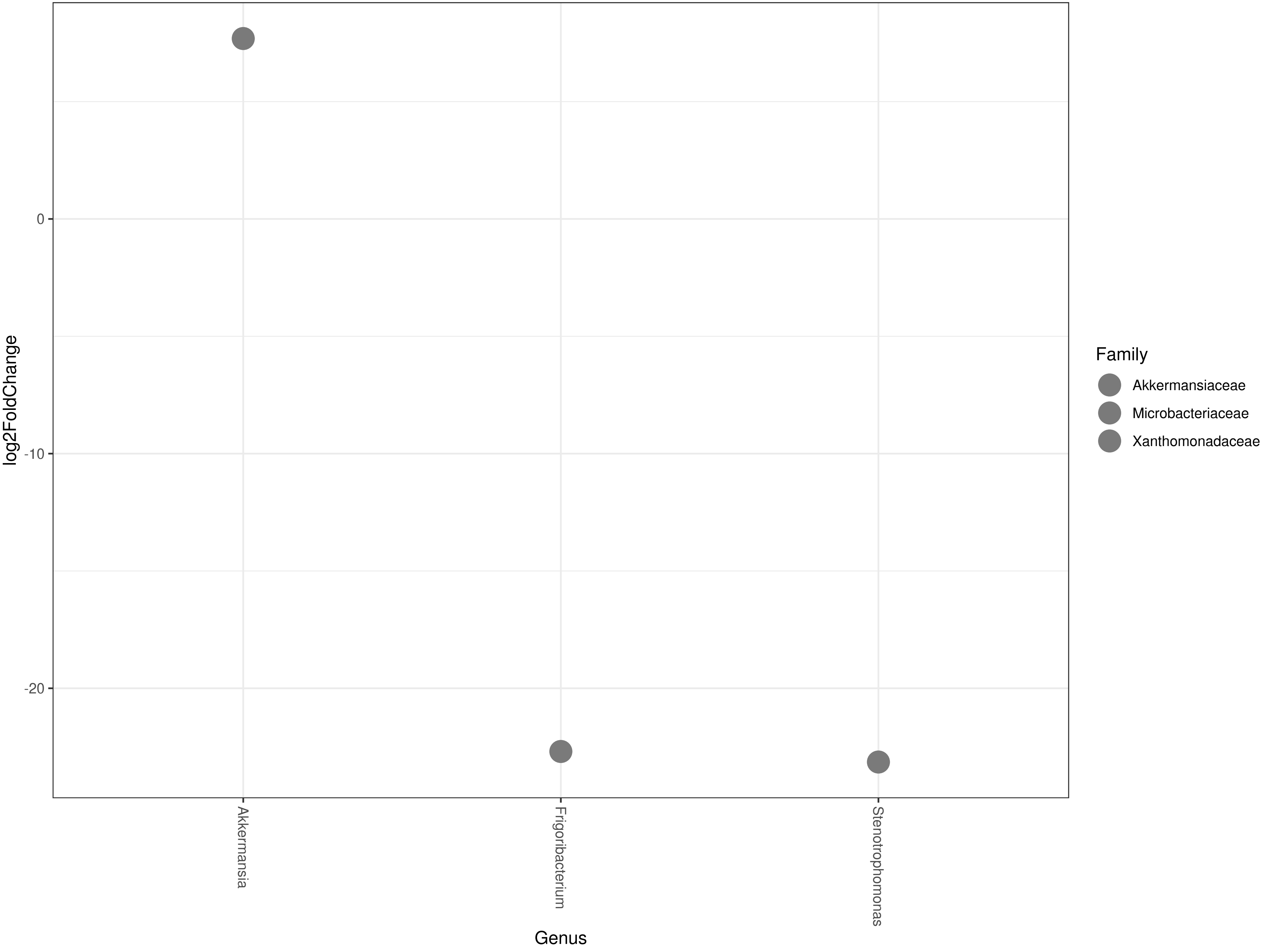

Supplementary figure 4: Differential abundance at time point 6 months (adj. p-value = 0.001)

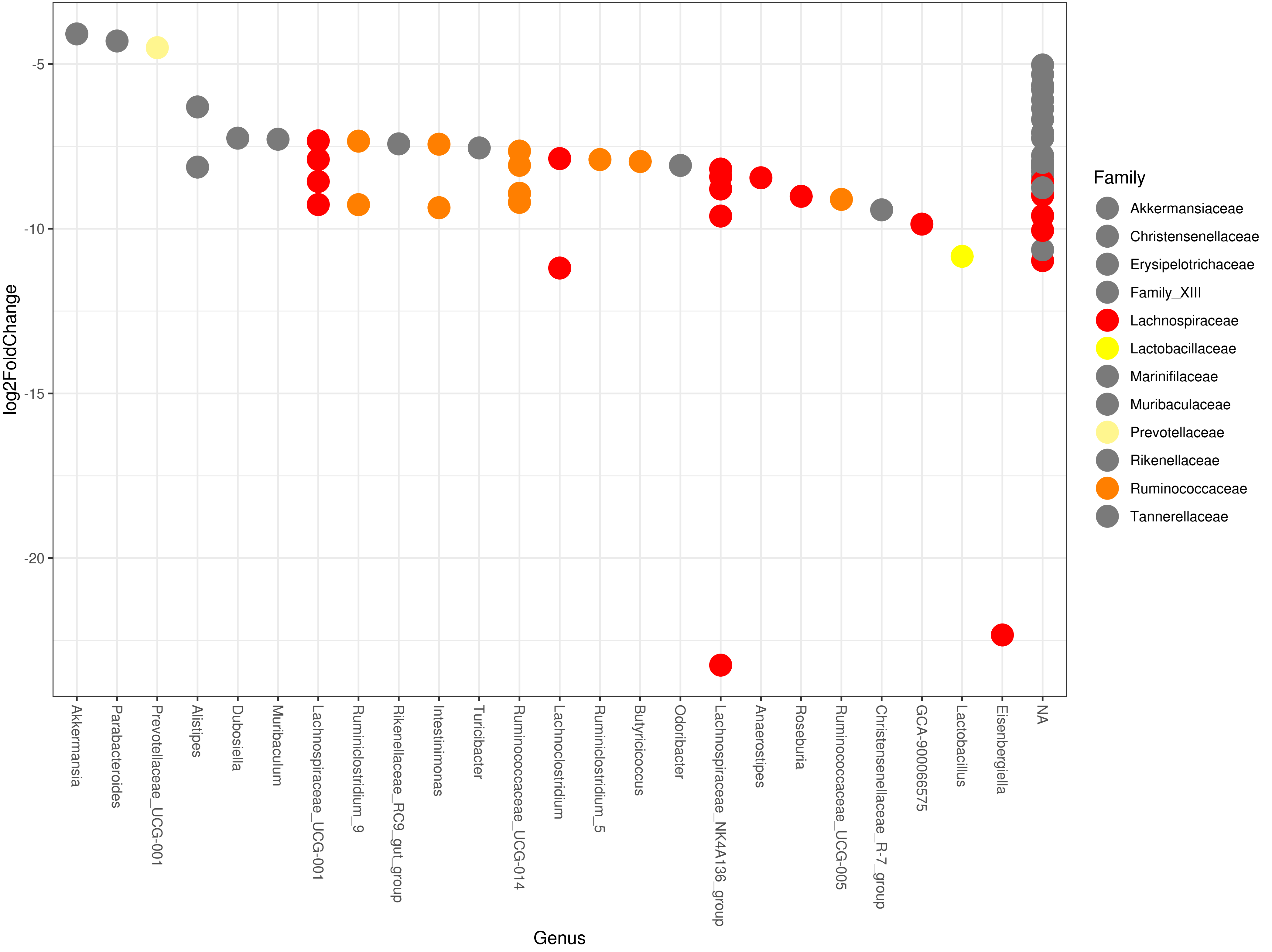

Supplementary figure 5 Differential abundance at time point 9 months (adj. p-value = 0.001)

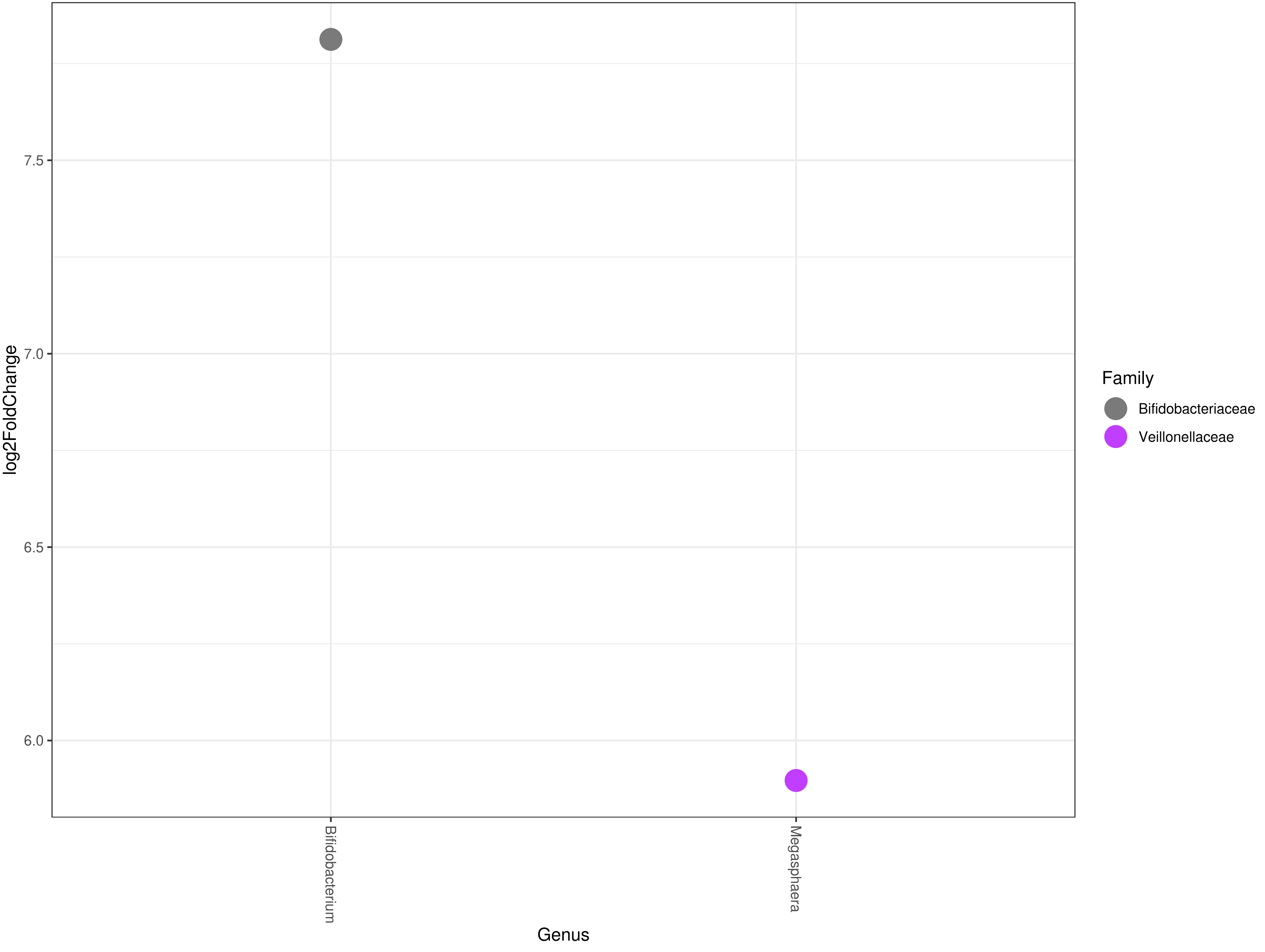

Supplement: Supplementary material 1 [file mgen-6-485-s001.pdf]
